# Genome-wide identification of *Azospirillum brasilense* Sp245 small RNAs responsive to nitrogen starvation and likely involvement in plant-microbe interactions

**DOI:** 10.1186/s12864-020-07212-7

**Published:** 2020-11-23

**Authors:** Vatsala Koul, Divya Srivastava, Pushplata Prasad Singh, Mandira Kochar

**Affiliations:** 1 The Energy and Resources Institute, Darbari Seth Block, India Habitat Centre, Lodhi Road, New Delhi, 110003 India; 2 TERI Deakin Nanobiotechnology Centre, Sustainable Agriculture Division, The Energy and Resources Institute, Gurugram-Faridabad Road, Gwal Pahari, Haryana 122003 India

**Keywords:** Sstress response, sRNA, *A. brasilense* Sp245, RNA-seq, PGPB, sRNAscanner

## Abstract

**Background:**

Small RNAs (sRNAs) are non-coding RNAs known to regulate various biological functions such as stress adaptation, metabolism, virulence as well as pathogenicity across a wide range of bacteria, mainly by controlling mRNA stabilization or regulating translation. Identification and functional characterization of sRNAs has been carried out in various plant growth-promoting bacteria and they have been shown to help the cells cope up with environmental stress. No study has been carried out to uncover these regulatory molecules in the diazotrophic alpha-proteobacterium *Azospirillum brasilense* Sp245 to date.

**Results:**

Expression-based sRNA identification (RNA-seq) revealed the first list of ~ 468 sRNA candidate genes in *A. brasilense* Sp245 that were differentially expressed in nitrogen starvation versus non-starved conditions. In parallel, in silico tools also identified 2 of the above as candidate sRNAs. Altogether, putative candidates were stringently curated from RNA-seq data based on known sRNA parameters (size, location, secondary structure, and abundance). In total, ~ 59 significantly expressed sRNAs were identified in this study of which 53 are potentially novel sRNAs as they have no Rfam and BSRD homologs. Sixteen sRNAs were randomly selected and validated for differential expression, which largely was found to be in congruence with the RNA-seq data.

**Conclusions:**

Differential expression of 468 *A. brasilense* sRNAs was indicated by RNA-seq data, a subset of which was confirmed by expression analysis. Four of the significantly expressed sRNAs were not observed in nitrogen starvation while 16 sRNAs were found to be exclusively expressed in nitrogen depletion. Putative candidate sRNAs identified have potential mRNA targets primarily involved in stress (abiotic and biotic) adaptability; regulation of bacterial cellular, biological and molecular pathways such as nitrogen fixation, polyhydroxybutyrate synthesis, chemotaxis, biofilm formation and transcriptional regulation. In addition to directly influencing bacteria, some of these sRNAs also have targets influencing plant-microbe interactions through adhesion of bacteria to plant roots directly, suppressing host response, inducing plant defence and signalling.

**Supplementary Information:**

The online version contains supplementary material available at 10.1186/s12864-020-07212-7.

## Background

The Alphaproteobacterial class of *Azospirillum* include physiologically distinct, rhizosphere competent, plant growth-promoting bacteria (PGPB) that promote the growth of a wide range of plants through diverse, direct and indirect mechanisms [1, 2]. Nitrogen fixation is not the most significant field function of this bacterial group, and it is the production of the plant growth regulators and their crosstalk which are the key signals for plant growth promotion effects [3–7]. Genome sequencing and analysis revealed that *Azospirillum* spp. which are now primarily established as plant-associated bacteria in the terrestrial habitat have actually transitioned from the aquatic environment [8, 9]. For improved adaptation in the rhizosphere, nearly 50% of its genome has been horizontally acquired from distantly related terrestrial bacteria [representatives of Rhizobiales (alpha-proteobacteria) and Burkholderiales (beta-proteobacteria)]. The horizontally transferred (HGT) genes consist of genes encoding cellulolytic activity (required for penetration into plant roots), attachment (using Type IV pili), and chemotaxis operon, among other essential genes required for plant-microbe interaction [8].

Specific genes involved in rhizosphere adaptation suggest niche-specific responses of the strains [9]. It is extremely important to study the extent of influence and regulation of these microbial plant growth regulators so that their effect on plants can be judged for their appropriate action and application. Basic knowledge about the modulation of key physiological properties of such PGPBs is crucial for understanding diverse aspects related to rhizosphere performance, modes of action and successful interactions with plant roots. Bacteria possess various regulatory mechanisms for stress adaptation and to improve the stress enduring capability, it is important to gather in-depth information of the underlying physiological and molecular mechanisms as well as unravel the participating intermediates especially during stress attacks [10–13]. The ensuing cellular cascade in response to changing environmental conditions is not always completely understood due to the lack of information about regulatory molecules such as small RNAs which play an essential role at the post-transcriptional level [14]. This knowledge will also help kindle ideas about how to advance the production and application strategies in PGPB inoculants.

In the last couple of decades, genome-wide searches in different bacteria have led to the discovery of a plethora of small, non-coding regulatory RNA molecules (sRNAs), which possess the capability to regulate expression of multiple genes, transcriptional factors, sigma factors, and chaperones, leading to profound effect on the cell physiology [14–17]. The bacterial sRNAs identified till now are heterogeneous in size and range from 40 to 550 bp, usually located in the intergenic regions and demonstrate a characteristic stem-loop secondary structure [18–22], though some exceptions exist [23]. A single bacterial genome is estimated to encode 200–300 sRNAs, possessing diverse functions such as plasmid replication [23], stress adaptation [24], regulating the expression of outer membrane proteins [25], iron homeostasis [26], quorum sensing [27] chemotaxis and biofilm formation [28], among others. Different studies have been carried out to discover sRNAs in plant-associated bacteria (PAB) like *Sinorhizobium meliloti, Bacillus subtilis, Bradyrhizobium japonicum, Azotobacter vinelandii,* and many others [29–34].

Azospirilla are the most well-known PGPB and consist of the strains Sp245, Sp7, and SM which are known to enhance the plant root morphology, specifically due to their two essential traits: biological nitrogen fixation (BNF) and biosynthesis of plant growth regulators (IAA, cytokinin, gibberellin, ethylene, and nitric oxide) [6, 35–37]. The genome size of *Azospirillum brasilense* Sp245 (GC content: 68.5%) is around 7.5 Mb distributed in seven replicons-main chromosome and six plasmids (AZOBR_p1-p6). Although its genome is sequenced, no study has been carried out to identify the sRNAs in this organism. The genome sequencing of *Azospirillum* sp. B510 revealed putative genes for two types of small RNAs: the B subunit of RNaseP (*rnpB*) and signal recognition particle RNA (*ffs*) [8], albeit no regulatory role has been associated with them so far.

In this study, we attempt to identify the sRNAs of *A. brasilense* Sp245 and quantify their transcription under nitrogen-limited conditions. Alongside genome-wide identification of novel sRNAs with follow-up Northern blots and qPCR, we provide a validated community resource for future studies. Many of these sRNAs may be involved in regulating important biological mechanisms such as plant growth regulators action, chemotaxis/motility, stress-responsive behaviour, nitrogen-metabolism, transcriptional regulation and plant association.

## Results

### Plant growth-promoting trait evaluation

Plant growth-promoting traits of *A. brasilense* Sp245 were investigated under various physiological conditions ranging from non-stressed to stressed nutrient conditions (predominantly carbon and nitrogen) to determine the variation in these traits which may be relevant in the rhizosphere niches for their functional behaviour. The results depicted in Table 1 indicate that polyhydroxybutyrate (PHB) production, NO production and Nitrogenase activity was significantly higher in both nutrient stress conditions in comparison to the non-stressed conditions while biofilm formation in nutrient stress conditions was not affected in strain Sp245. IAA production on the other hand was negatively influenced in both the nutrient stress conditions.

**Table 1:** Biochemical parameters of *Azospirillum brasilense* Sp245 under various physiological conditions ranging from non-stressed to stressed conditions reflecting the physiological competence of the strain

Set	Physiological conditions	μg IAA/OD (IAA production)	μg PHB/mg cell biomass (PHB accumulation)	OD_560_ per cellular OD_560_ -biofilm units (Quantitative Biofilm formation)	nmol/h (Ethylene Production, Nitrogenase activity)
N1	C + N	16.27 ± 0.046	11.3 ± 0.2	11.94 ± 1.26	0.05
N2	C/2 + N/2	10.02 ± 0.053 ^a^	54.2 ± 0.4 ^a^	13.02 ± 1.86	0.72 ^a^
N3	C + N/2	14.34 ± 0.028 ^a^	42.4 ± 0.4 ^a^	13.68 ± 0.98	0.75 ^a^
N4	C-N				0.39 ^a^

The values represent the average of three independent sets ± standard error from the mean

Statistical analysis was performed as described in Materials and methods

^a^, Values significantly different from strain Sp245 grown in set N1 at full strength C + N at *P* ≤ 0.05

IAA was quantified as mentioned previously. 1 mM Trp was added to each culture at the time of subculture and the respective substrates were added after 6 h of growth

### sRNA sequencing and prediction

RNA samples with high RIN value for VC (RIN 10) and VN (RIN 9.5) were used for RNA-seq. The cDNA libraries were prepared and proceeded for sRNA sequencing on an Illumina NextSeq500 platform (Illumina, USA). QC of the raw data files (VC_R1, R2 and VN_R1, R2) has been given in Additional File 1. The processed output files were aligned to the reference genome using Bowtie v2.1.0 [38]. During the alignment, in VC 100% of the 5,513,335 reads were paired, out of which 4.57% were aligned concordantly zero times, 83.97% were aligned concordantly exactly one time and 11.46% were aligned concordantly more than one times. The total alignment rate obtained for VC and VN was 97.47 and 97.07% respectively. The summary of the outcome of each step of QC and differential expression analysis is shown in Table 2. By using Cufflinks, a total of *n* = 3860 transcripts with a difference in expression levels were predicted in the two samples (Additional File 2) (Old AF4), of which *n* = 468 had length between 50 and 500 bp under control and nitrogen-stress conditions (VC and VN, respectively). *N* = 59 sRNAs showed significant differential expression (FDR corrected *p*-value 0.05) and also had transcript length between 50 bp and 500 bp. and thus were further analysed for prediction of potential mRNA targets (Additional File 2). Of these sRNAs, 41 were up-regulated and 18 were downregulated in VN in comparison to VC (Table 3). The secondary structures prediction by Mfold for these 59 significantly expressed sRNAs revealed that all of them attained the complex stem-loop conformations which are characteristic of known bacterial sRNAs. These 59 sRNAs were further annotated and investigated for potential mRNA targets to understand the important pathways regulated by them under nitrogen stress.

**Table 2:** Summary of sRNA sequencing analysis of *Azospirillum brasilense* Sp245

S.No.	Steps	VN	VC
1	Total number of raw sRNA sequence reads	R1: 27200643	R1: 16557793
		R2: 27200643	R2: 16557793
2	Total number of Reads after Adapter removal and Quality filtering at q20	R1: 7731698	R1: 5513335
		R2: 7731698	R2: 5513335
3	Mapping percentage with reference genome (*Azospirillum brasilense* Sp245)	97.07%	97.47%
4	Number of assembled reads (sRNA)	15,614	15,424
5	Assembled sRNAs sorted based on size 50-500 bp	3838	3788
6	Total number of differentially expressed sRNAs in both samples VN & VC (Additional File 2)	3860	
7	No. of differentially expressed sRNAs, between 50 and 500 bp length, (Additional File 2)	458	
8	No. of significantly differentially expressed sRNAs *p*value: < 0.05 (with FDR) & sRNAs length between 50 and 500 bp (Additional File 2)	59	
9	No. of Downregulated sRNA genes (Table 3)	18	
10	No. of Upregulated sRNA genes (Table 3)	41	

GenBank Assembly accession used for Bowtie: GCA_000237365.1

VC and VN imply strain Sp245 cells grown in SSM with the full strength of nitrogen and SSM with half strength of nitrogen, respectively

**Table 3:** Differentially expressed sRNAs in controlled nutrient conditions (VC) versus nitrogen stress conditions (VN) in *Azospirillum brasilense* Sp245

Upregulated sRNAs					Downregulated sRNAs				
sRNA_id	sRNA size	VC1 value	VN1 value	*p*_value	sRNA id	sRNA size	VC1 value	VN1 value	*p*_value
*AbSp_118*	225	0	37.8556	0.00005	*AbSp_119*	225	35.0559	2.83011	0.04805
*AbSp_136*	241	0	20.542	0.0019	*AbSp_124*	228	4.43713	0	0.0344
*AbSp_155*	255	43.9049	207.732	0.0484	*AbSp_149*	252	98.7514	1.16048	0.00055
*AbSp_177*	270	0	5.37379	0.039	*AbSp_159*	258	1100.41	9.23603	0.0018
*AbSp_189*	279	18.3086	556.103	0.00255	*AbSp_160*	258	5.46297	0	0.01445
*AbSp_19*	90	0	21.5451	0.02095	*AbSp_178*	270	25.8129	4.6556	0.04725
*AbSp_193*	282	42.0717	3283.41	0.00035	*AbSp_220*	300	68.0688	10.5244	0.04105
*AbSp_194*	282	630.942	7287.28	0.02545	*AbSp_252*	324	2.8567	0	0.0344
*AbSp_2*	74	19,184.8	224,650	0.0091	*AbSp_264*	330	58.9137	9.8017	0.031
*AbSp_214*	297	45.2875	397.939	0.03035	*AbSp_293*	363	237.893	30.1123	0.03395
*AbSp_219*	300	0	5.14205	0.0208	*AbSp_332*	396	21.2397	3.05772	0.01735
*AbSp_239*	315	20.0026	515.628	0.0033	*AbSp_382*	441	259.241	38.0665	0.0494
*AbSp_257*	326	58.2017	847.189	0.009	*AbSp_39*	183	10.8689	0	0.01445
*AbSp_268*	333	22.4175	921.357	0.0015	*AbSp_391*	450	39.8958	3.30199	0.00575
*AbSp_29*	165	27.5982	1100.28	0.00495	*AbSp_410*	462	141.891	15.8314	0.0264
*AbSp_294*	363	0	2.71895	0.039	*AbSp_450*	498	26.0091	3.96386	0.04145
*AbSp_308*	375	46.3089	1225.36	0.0029	*AbSp_51*	189	75.8142	12.4539	0.0439
*AbSp_313*	379	25.3495	654.802	0.00445	*AbSp_73*	201	9514.16	639.783	0.0073
*AbSp_314*	381	0	3.1861	0.00355					
*AbSp_324*	387	42.9103	368.276	0.023					
*AbSp_336*	402	18.0015	155.195	0.0307					
*AbSp_343*	408	98.3318	595.871	0.04125					
*AbSp_345*	411	3.53559	30.7108	0.04255					
*AbSp_347*	414	253.799	2065.6	0.04155					
*AbSp_355*	420	5.51417	255.763	0.0127					
*AbSp_359*	423	0	4.64154	0.0114					
*AbSp_38*	183	453.436	8048.83	0.01165					
*AbSp_390*	450	6.91909	1152.3	0.0025					
*AbSp_409*	462	4.64182	137.132	0.0083					
*AbSp_419*	471	12.6331	220.605	0.0047					
*AbSp_445*	492	0	9.78685	0.00165					
*AbSp_449*	498	20.2349	841.26	0.00065					
*AbSp_45*	186	0	193.89	0.0001					
*AbSp_58*	192	0	117.01	0.0001					
*AbSp_59*	192	0	10.2805	0.006					
*AbSp_64*	195	0	11.1888	0.006					
*AbSp_65*	195	0	3.64177	0.02095					
*AbSp_66*	195	0	4.41882	0.02095					
*AbSp_67*	195	8.7278	108.935	0.0322					
*AbSp_8*	76	3224.96	44,002.6	0.01065					
*AbSp_93*	210	0	7.97932	0.0208					

Out of the total predicted 3860 candidates, 12 sRNAs were randomly selected and used in the validation process by northern blot analysis with probes mentioned in Additional File 3. It was observed that the selected 12 sRNAs were expressed under both the non-stressed (control conditions (VC) as well as nitrogen stress-induced conditions (VN). Apart from conforming to the known sRNA parameters (such as location and size), they possessed high FPKM values since that would make their detection possibly easy. Northern blot analysis revealed that all 12 sRNAs showed a single band (Additional File 3).

### *In-silico* identification of novel sRNAs

In parallel to the RNA-seq analysis, sRNAscanner was used to scan the multi-replicon genome of *A. brasilense* Sp245 (GenBank Assembly accession: GCA_000237365.1) to predict putative sRNAs. Two sRNAs labelled AbSp_464 (location: AZOBR_p4; NC_016596, sRNA locus: NC_016596.1:167096–167,348) and AbSp_465 (Location: AZOBR_p6; NC_016597 sRNA locus: NC_016597.1:36503–85,307) were predicted (Table 4). Both the computationally predicted sRNAs, AbSp_464 and AbSp_465 were also validated by Northern blotting **(**Additional File 3).

**Table 4:** Details of the sRNAs predicted by in silico analysis with sRNAscanner

sRNA	Genomic Location	Position	Sequence (5′-3′)	Length (bp)
AbSp_464^a^	Plasmid, AZOBR_p4	NC_016596.1:167096–167,348	TGTGTTTTAAATGGATGTGCGAATTCTATTGTTTGTTATCAGCGGTTTG	49
AbSp_465^a^	Plasmid, AZOBR_p6	NC_016597.1:36503–85,307	TGACAATGCAAAGACCGTCTCCTTTTCCGCAATCAAACAAGAAGAAAATGCCCTCGCTCTATTCCTACACTCGGTGAGCGCTGTTCCCGTCGAGCTGGCAGCC	103

^a^These sRNA were also identified through RNA-seq analysis

### Annotation of sRNAs

For preliminary functional annotation, the sRNA sequences were searched against the Rfam database and BSRD. Rfam database is a collection of RNA families comprising of non-coding RNA genes, structured cis-regulatory elements and self-splicing RNAs, while BSRD is a repository for bacterial small regulatory RNAs. A total of 4 of the 59 uncovered sRNAs in our study showed homology with previously reported sRNAs in BSRD while 2 entries show homology to the tRNA family in Rfam (Table 5, AbSp_39, AbSp_308, AbSp_345, AbSp_93, AbSp_2, AbSp_8), which also supports the finding that all the other identified sRNAs are likely to be novel.

**Table 5:** *Azospirillum brasilense* Sp245 sRNAs with homologs in BSRD and homology with Rfam families

sRNA Id	sRNA gene ID	Subject	Strain name	Score	E-value	Alignment Length / homology (%)	Function
AbSp_39	AZOBR_RS26955	sgur2698.1	*Geobacter uraniireducens* Rf4	34.2	0.036	100	Cyclic di-GMP-II riboswitch
AbSp_308	AZOBR_RS37495	scch1500.1	*Chlorobium chlorochromatii* CaD3	36.2	0.021	100	Regulatory element
AbSp_345	AZOBR_RS30660	ssme817.1	*Sinorhizobium meliloti* 1021	36.2	0.019	100	cis-encoded antisense RNA
AbSp_93	AZOBR_RS24525	seco498.1	*Escherichia coli* K12 MG1655	50.1	7.00E-07	96	Trans-encoded antisense RNA
AbSp_2	AZOBR_RS36450	RF0000	-NA	63.1	1.20E-14	100	tRNA family
AbSp_8	AZOBR_RS18695	RF00005	-NA	60.2	7.90E-14	100	tRNA family

### Conserved motifs and promoter prediction

All 59 sRNA sequences were used for motif prediction using MEME at default parameters, and the top 3 motifs were selected based on high score, length (> 9 nucleotides), and *p*-value (< 1e-10). The results indicated that sRNAs share common motif sequences (Additional File 4). Searching these motifs in the motif database using a comparison tool, TOMTOM [39] revealed that motif M1 possessed homology with the transcriptional regulator AlgR which controls a variety of virulence factors, including alginate production, twitching motility, biofilm formation, and hydrogen cyanide production in *Pseudomonas aeruginosa* [40]. Motif M2 shares similarity with *Escherichia coli* RutR which is the master regulator of genes involved in pyrimidine catabolism [41] while motif M3 was homologous to the motif present in the transcriptional factor, *Pseudomonas* sigma regulator (PsrA) involved in bacterial epiphytic fitness, quorum sensing and plant interactions [42].

BPROM [43] predicted the presence of one promoter each in the upstream region of 19 sRNAs, while no promoters could be predicted for other significantly expressed sRNAs **(**Additional File 4). Additionally, AbSp_465 revealed the presence of a RpoD17 binding site at position 96 for the promoter predicted at position 77. For AbSp_459 presence of RpoD19 and RpoD17 binding sites were observed at positions 570 and 574, respectively for the promoter predicted at position 588. For other sRNAs with predicted promoters, no known sigma-factor binding sites (within 500 bp upstream region) were observed for their promoters.

### Prediction of target mRNA

**Fig. 1:**
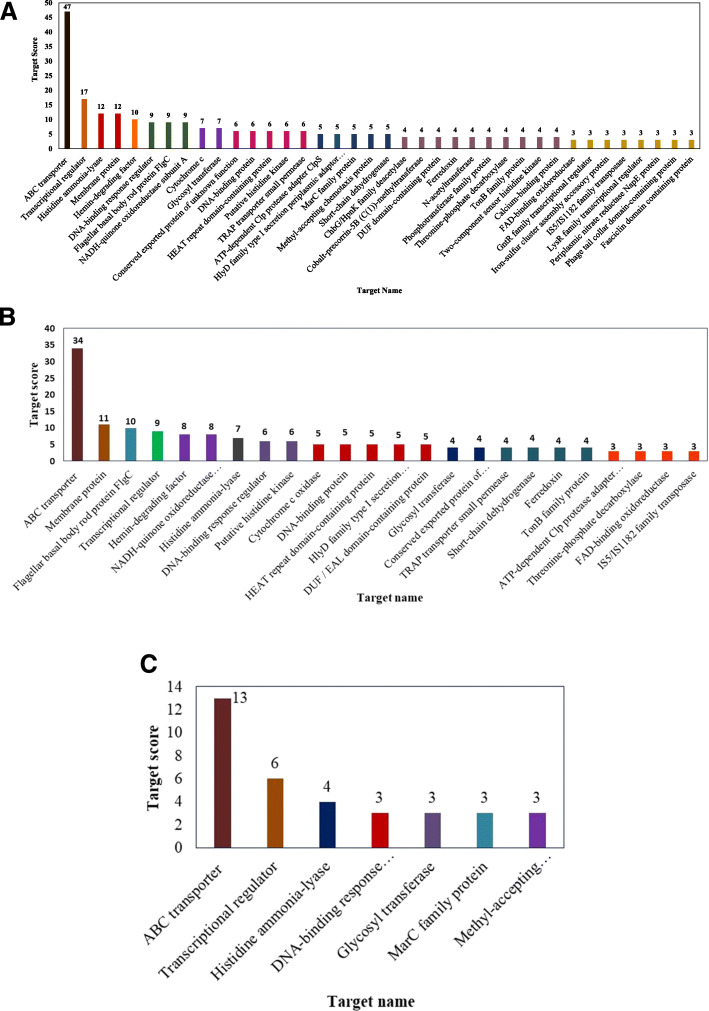
Targets predicted for each of the 59 candidate sRNAs identified in this study (**a**). The gene-class and frequency of putative mRNA targets relevant for plant-microbe interactions was compared between sRNA candidates with upregulated expression in VN (**b**) and downregulated expression in VN (**c**). For ease of graphical representation, only those targets with score ≥ 3 are shown. For further details please see Additional File 5

Target prediction for the selected 59 sRNAs was done using IntaRNAv2.0 [44]. In total, 56 different gene targets were predicted (which fall under different functional categories) for the 59 sRNA candidates (Additional File 5). Many of these targets (~ 20) are known to be involved in plant-microbe association and interactions at varying levels. Some of the gene targets were significantly frequent among the differentially expressed 59 sRNAs (Fig. 1a). The highly enriched category included ABC proteins, which included ABC transporter proteins, ATP-binding protein, ABC transporter permease, ABC transporter substrate-binding protein, Amino acid ABC transporter permease, Amino acid ABC transporter substrate-binding protein, iron ABC transporter permease and iron ABC transporter substrate-binding protein and was commonly predicted for 31 of the identified 59 sRNA candidates. The next prevalent category consisted of transcriptional regulators including AbrB, AsnC, XRE, GntR, IclR, LysR, MarR, MarC, TetR/AcrR families of transcriptional regulators and was shared between 15 candidate sRNAs. The gene-class and frequency of putative mRNA targets relevant for plant-microbe interactions were compared between groups of sRNA candidates with (a) upregulated expression in VN (Fig. 1b) and (b) down-regulated expression in VN (Fig. 1c). ABC transporters, transcriptional regulator proteins, Histidine based regulatory proteins (including lyase, kinases response regulators etc.), DNA binding response regulator and glycosyltransferases were commonly shared target protein families between the sRNAs that were either upregulated or down-regulated on exposure to nutrient stress in sample VN. Noticeably, targets of preferentially downregulated sRNAs included the transcriptional regulator protein MarC, methyl-accepting chemotaxis protein and DUF-domain (EAL domain) containing proteins were preferentially predicted for the group of sRNAs showing significantly downregulated expression in VN.

On the other hand, targets favourably predicted for the upregulated sRNA in VN included the flagellar basal body rod protein, membrane protein and hemin-degrading factor (Additional File 5). This could imply towards increased motility under nitrogen stress conditions leading to improved colonization of host plant roots and better establishment of the bacterium in the root niches. With the target flagellar basal body rod protein FlgC, AbSp_2 and AbSp_93 could negatively regulate the motility of *A. brasilense* by regulating flagella structural components. A similar response has been observed in *Salmonella enterica* [45].

**Fig. 2:**
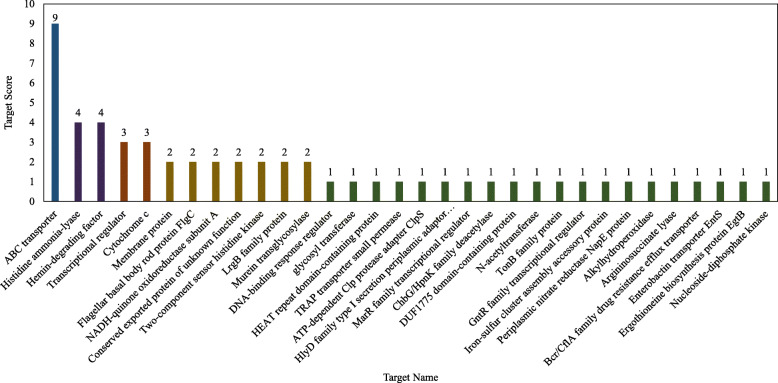
Target prediction output for the 14 of the 59 significantly differentially expressed candidate sRNAs from non-protein coding loci of *A. brasilense* Sp245 suggested ABC transporter proteins, histidine ammonia lyase, transcription regulators and hemin degrading factor as the prominent targets

**Fig. 3:**
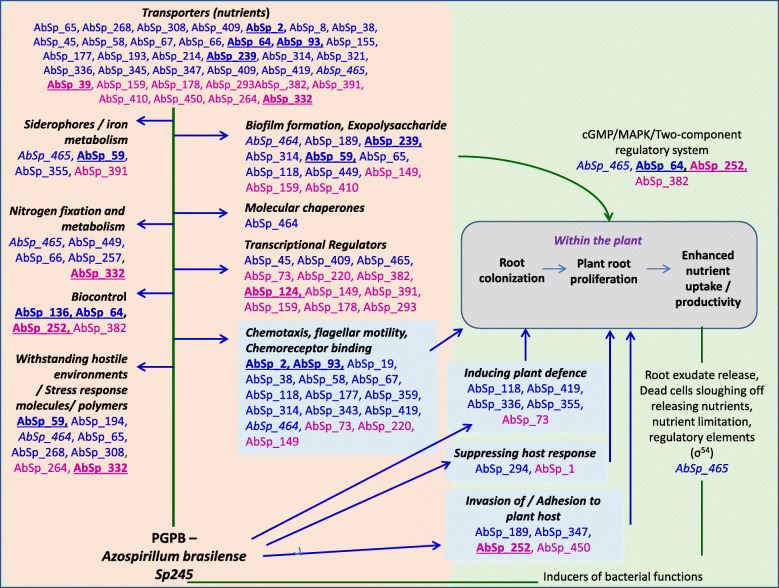
Model highlighting ~ 60 sRNAs from *A. brasilense* Sp245 with predicted targets likely to influence plant association, colonization, productivity as well as regulation of various essential bacterial biological networks and plant growth influencing functions. sRNAs with upregulated targets are highlighted in blue while those that have downregulated targets are indicated in pink. The sRNAs from non-protein coding loci are underlined

Fourteen of the 59 significantly differentially expressed candidate sRNAs were coded by non-protein-coding genes as no InterProScan search hits were found (Additional File 6). From these 14 sRNAs, a large proportion may likely be novel sRNAs with no known sRNA homology. Target prediction output for sRNAs from non-protein-coding loci of *A. brasilense* Sp245 suggested ABC transporter proteins, histidine ammonia-lyase, transcription regulators and hemin degrading factor as the prominent targets (Fig. 2, Additional File 6). Interestingly, 6 of these were upregulated while 8 were down-regulated in nitrogen stressed conditions in *A. brasilense* Sp245. These may likely be involved in the nutrient stress response and consequent regulation of the bacterial physiology. Altogether, the sRNA targets prediction suggested possible comprehensive involvement of the sRNAs in the regulation of various essential bacterial biological networks and plant growth influencing functions such as those mentioned in Fig. 3. Functional evaluation of a selected few candidate sRNAs is being taken up in our lab and may also be the focus of future studies of different research groups.

### Validation of differential expression of sRNAs

**Fig. 4:**
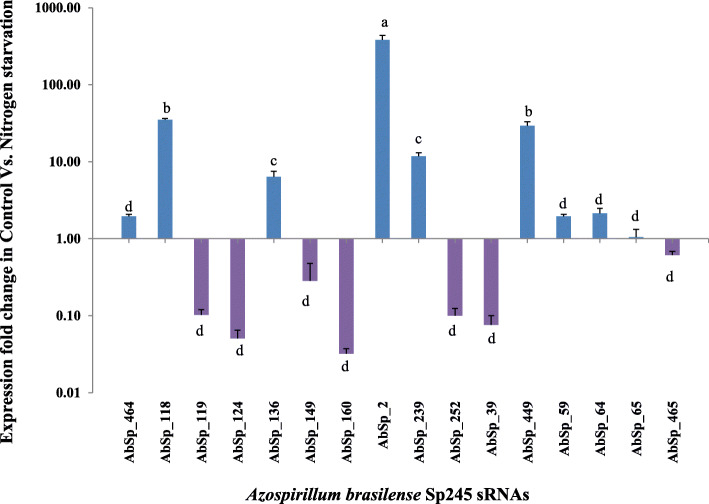
Differential expression validation of selected sRNAs by quantitative real time PCR. The mean of normalized expression fold change data of 3 replicates is reported along with the standard error from mean. The data was statistically analysed for variance by ANOVA followed by Duncan’s Post hoc analysis at *P* ≤ 0.05. Note: ^a, b, c, d^ reflect the significance in sRNA expression in nutrient starvation at *P* ≤ 0.05

Sixteen sRNAs out of a total of 59 were selected for validation by quantitative RT-PCR. These sRNAs were selected based on (i) highly significant differential expression between VC and VN (unstarved versus nitrogen starved conditions) or (ii) role in the regulation of important mRNA targets. The selected sRNAs and primers designed are shown in Additional File 7 and the results of differential expression analysis are shown in Fig. 4. Generally, the trend observed with RNA-seq data was also observed with the qRT-PCR analysis. Further, the 4 sRNAs (AbSp_124, AbSp_160, AbSp_252 and AbSp_39 did show very low or almost negligible expression along with the downregulated AbSp_119 and AbSp_149 sRNAs. AbSp_118, AbSp_136, AbSp_2 and AbSp_449 were found to be significantly expressed in nitrogen starvation response of *A. brasilense* Sp245. Other upregulated nitrogen starvation responsive sRNAs were AbSp_59, AbSp_64, AbSp_65, and AbSp_464 (Fig. 4). AbSp_465 did not show a significant fold change in expression even though RNA-seq data seemed to indicate that it is nitrogen starvation responsive sRNA This warrants further investigation and may be due to an interplay with other regulatory molecules.

## Discussion

In order to survive in the rhizosphere, the microorganisms need to constantly adapt to the changing environment. These environmental conditions, for example, change in temperature, nutrition, pH, salt concentration etc., may sometimes be favourable or otherwise pose a challenge and induce stress responses in the bacteria [46]. The IAA produced in nutrient stressed conditions by strain Sp245 have been observed to be significantly lower while the nitrogenase activity and PHB produced by the strain were significantly higher than in unstressed conditions. As such the IAA and PHB production is known to be regulated or enhanced in nutrient deficiency in *A. brasilense* Sp245 [47, 48]. The bacterial defence mechanism is usually a cascade of events with multi-layer regulation. The ensuing cellular cascade in response to environmental stresses is not always completely understood due to insufficient information of regulatory molecules which play an essential role at the post-transcriptional level. The cumulative impact of all the members of the complex regulatory network enables the bacterial cells to adapt and survive under a dynamic environment [24, 49]. Various studies have exploited the RNA-seq approach to reveal previously undetected small RNAs across numerous bacterial and cyanobacterial species including strains of *Pseudomonas aeruginosa*, *P. putida* and *P. syringae* [50, 51], *Neisseria gonorrhoeae*, *Salmonella enterica* and *Streptococcus pyogenes* [52–55], *Vibrio cholerae* [56, 57], and *Synechocystis* sp. PCC6803 [58]. A genome-wide search for novel regulatory RNAs and identified sRNA RyhB, in the model organism, *E. coli*, which was later, found to regulate the expression of genes in iron homeostasis [25, 26]. The efficiency and sensitivity of the existing sRNA prediction tools can be improved by suitably modifying their algorithm in accordance with the bacterial genome of interest. A recent exercise of modulation of existing sRNA prediction tools with *Agrobacterium* strains led to the prediction of 384 sRNAs [59]. We thus used a combinatorial approach involving RNA-seq and computational tools for genome-wide detection of sRNAs in the *A. brasilense* Sp245 bacterial genome. The RNA-seq data analysis indicate the presence of many novel sRNAs in *A. brasilense* Sp245. Additionally, 2 sRNAs (AbSp_464 and AbSp_465) were also recovered through *in-silico* analysis. The identification of both these sRNAs from RNA-seq data is note-worthy.

Differential RNA-seq (dRNA-seq) led to the identification of genome-wide transcriptional start sites (TSSs) and differentially expressed genes in gram-positive PGPR *Bacillus amyloliquefaciens subsp. plantarum* FZB42, grown under different rhizosphere-mimicking conditions (in the presence of root exudate and soil extract). This study also revealed 86 sRNA candidate genes, of which 13 were previously known sRNAs [34]. Out of 86 potential candidates, only 19 candidates could be detected as distinct transcripts. Whole-genome survey of *Sinorhizobium meliloti* by deep-sequencing revealed that 3% of its genes code for trans-encoded sRNAs [60]. A study using nutrient starvation stress response as the key physiological indicator in *Salmonella enterica* SL1344 and confirmed that 63 sRNAs are differentially expressed in this bacteria in different growth conditions [53]. Out of these, many are involved in varying degrees in the carbon-starvation stress response of the bacterium. In lines with these results, our study also reports the discovery of ~ 468 differentially expressed candidate sRNAs from non-stressed vs. nitrogen-starved *A. brasilense* Sp245 cells. Of these, 59 sRNAs are differentially expressed (41 sRNAs upregulated and 18 downregulated sRNAs) with 16 sRNAs being exclusively expressed in nitrogen starvation and 4 sRNAs not being expressed in nitrogen depletion conditions. 53 of the 59 are potentially novel sRNAs as they bear no BSRD or Rfam homology and 16 have been functionally validated for differential expression.

To identify the biological function of the bacterial sRNAs, use of an *in-silico* approach for target prediction is commonly conducted to recognize potential mRNA targets and thereby the regulatory networks influenced by the sRNA. *P. aeruginosa* has been shown to possess iron-responsive sRNAs, PrrF1 and PrrF2 [61], and heme-responsive sRNA, PrrH [62]. This study employed an approach similar to ours, to determine potential targets of PrrH and subsequently, its involvement in virulence was hypothesized. The regulatory RNAs in *Actinobacillus pleuropneumoniae* were predicted using multiple *in-silico* tools and post their experimental validation; the possible mRNA targets were investigated to identify the pathways in which the sRNAs might be functional [63].

It is likely that *A. brasilense* candidate sRNAs of the *marC* target gene may be involved in the stress response regulation of *A. brasilense*. DUF or the GGDEF domains are involved in processing cyclic-di-GMP, a universal bacterial second-messenger molecule [64, 65]. Cyclic-di-GMP is critical to the regulation of key bacterial functions are motility, chemotaxis, capsular polysaccharide formation, biofilm formation and cellulose synthesis [66]. The *Azospirillum* genomes encode a large number of proteins that are likely involved in cyclic-di-GMP metabolism and these together regulate host specificity and environment-specific adaptation of the bacteria in the soil niches. Whether cyclic-di-GMP physiologically regulates the *mcp* gene and other genes involved in chemotaxis is yet to be seen. Further studies would be needed to confirm this.

Located between a metallo-dependent amidohydrolase and the sulphite exporter TauE (also a DUF family protein), the probable mRNA targets of AbSp_136 were protein export membrane protein, enterobactin transporter, hemin-degrading factor and the LgrB family protein. Enterobactin is a string Fe (III) chelator thereby helping bacteria scavenge Fe and hydroxyl groups (hydrolyzed enterobactin) not only to meet their critical requirement but simultaneously reduce oxidative stress [15]. LgrB is a paralog of the *lgrE* gene (large GC rich genes belonging to the DUF family) which is related to Nucleotide-binding Oligomerization Domain (NOD) containing proteins involved in regulating apoptosis and phytopathogen resistance (R genes) in plants.

Overall, the model presented in Fig. 3 highlights the sRNAs from *A. brasilense* Sp245 which have predicted targets with the potential to influence plant association, colonization, productivity as well as modulation of bacterial and plant physiology in plant-microbe association conditions. These include nitrogen-fixation and utilization, siderophores and iron homeostasis, biofilm formation, exopolysaccharide production, nutrient transporters, high-temperature stress adaptability, capability to withstand hostile environments and stress responsive behaviour (including the carbon storage polymer, poly hydroxyl butyrate production), chemotaxis/motility, adhesion to plant roots, induction of plant defence and suppression of plant response and transcriptional regulation. There are possibly over 60 sRNAs with likely involvement in plant association and growth promotion alongside plant-microbe interactions. It is also likely that many of the candidate sRNAs that are not differentially expressed in nutrient starvation conditions may still have a regulatory role in plant-microbe interactions and traits relevant to the same, either directly or through Hfq - pairing or antisense base – pairing. It is hence a strong possibility that further experimental evidence may confirm that the number of sRNAs involved in *Azospirillum*-plant interaction may be even greater than those identified in this study.

The eubacterial sRNAs generally do not contain expressible ORFs, although some of them, such as SgrS or RNAIII of *Escherichia coli* or *Staphylococcus aureus*, also contain coding regions [16]. It may also be the case with some of the sRNAs identified in this study from *A. brasilense* Sp245. The characteristic stem-loop, secondary structure of the sRNAs protects the molecule and essentially its binding sites from RNase degradation within the cellular environment. The presence of rho-independent terminators is a well-documented phenomenon for Hfq-dependent sRNAs [67–69], and only in case of 3 sRNAs terminator sequences could be predicted, suggesting the dependence of these 3 sRNAs on the RNA chaperone (Hfq, AbSp_67, AbSp_149 and AbSp_159). For the remaining sRNA either independence of Hfq or the presence of some additional mechanism for Hfq-sRNA interaction may well be observed when studied in greater detail.

The bacterial sRNAs were initially not known to possess recurrent or conserved nucleotide motifs [70], however, recent studies have revealed that certain sRNA families contain protein binding motifs to inhibit the interaction of the protein with their target mRNAs. Also, sRNAs possessing a common motif in their binding sites suggest them to have similar target mRNAs [16]. The function of the sRNA can primarily be judged by the presence of conserved motifs and the promoters. The motif prediction of the 59 significantly expressed sRNAs revealed that they shared common motif sequences (M1, M2 and M3), which possessed homology with essential bacterial transcriptional regulators which influence cellular mechanisms such as chemotaxis, biofilm formation, quorum sensing, plant interaction, virulence, among many others [71]. The presence of such master transcriptional regulation motifs corroborated the role of sRNAs identified in our study as putative global regulators which may influence essential cellular cascades in the physiology and function of plant growth-promoting bacteria.

The sRNA promoters are sensitive to environmental fluctuations and regulate the sRNA expression in response to stress [14]. A recent study has revealed that the properties of the sRNA promoter influence the negative feedback circuit existing between two-component systems, EnvZ-OmpR and the sRNAs OmrA/B [72]. The promoter prediction uncovered the presence of one promoter each in the upstream region of 19 sRNA sequences, and sRNAs AbSp_465 and AbSp_459 possessed RpoD binding sites. RpoD is a primary sigma factor expressed during exponential growth and preferentially transcribes genes associated with fast growth, such as ribosomal operons and other protein-synthesis related genes. Further studies corroborating the functional characterization of the sRNAs are required to establish and enhance our understanding of the underlying mechanism of stress adaptability in *Azospirillum* and its interaction with various host plants.

## Conclusions

Over the years the sRNA identification in PAB and their expression study under different growth conditions, and their functional characterization have improved the knowledge of bacteria-host interactions. We have integrated an RNA-seq and in silico sRNA identification approach to generate the first list of candidate sRNAs in the well-known PGPB, *A. brasilense* Sp245. These have been curated in non-starved and nitrogen starved conditions and observations made point towards the regulatory role of the identified and validated sRNAs in *A. brasilense* Sp245 in not only bacterial stress tolerance but also plant-microbe association and interactions leading to plant growth promotion. The comprehensive analysis of the candidate sRNAs presented in this paper highlights the existence of functionally important sRNAs in non-stressed and nitrogen starvation conditions in *A. brasilense* Sp245. This research will stimulate further work in the field of PGPB, improvement of their efficacy and subsequent development of improved *Azospirillum*-based bioformulations which could further reduce the use of chemical fertilizers and enhance the productivity of the associated plants exposed to abiotic stress.

## Methods

### Bacterial strain, media and growth conditions

*A. brasilense* Sp245 (obtained from Prof. Gladys Alexandre, The University of Tennessee, USA) was used in this study. The bacterial culture was maintained on Luria–Bertani agar with 50 μg/ml ampicillin. The purity of the culture was checked periodically on modified nitrogen-free basal (OAB) medium as described [73]. For all experiments, an initial OD_560_ of 0.1 (unless otherwise stated, ~ 2 × 10^7^ cfu/ml) was maintained by subculturing the overnight-grown culture of strain Sp245 (18 h) in 20 ml of buffered standard succinate medium (SSM) [74]. The cells were grown at 30 °C, 180 rpm for all experiments.

### Plant growth-promoting trait evaluation

The PGPR traits relevant for plant growth were evaluated by quantifying the plant growth regulator, IAA; nitrogenase activity, poly-β-hydroxybutyrate (PHB) production and biofilm formation. IAA produced by the bacteria was quantified according to [6, 75]. The three conditions were: SSM with full strength Carbon and Nitrogen (C + N; N1); SSM with half strength Carbon and Nitrogen (C/2 + N/2; N2); SSM with full strength Carbon and half strength Nitrogen (C + N/2; N3) in presence of the precursor, 1 mM L-Trp. To account for the variation caused by the growth, IAA values were normalized to the cell density (OD) in each set and were thus represented as μg IAA/OD.

Quantification of PHB production was carried out indirectly based on the principle that the hydrolysis of PHB using concentrated H_2_SO_4_ produces crotonic acid which can be easily quantified by HPLC [76]. Since the production of PHB is maximum during stationary phase, overnight grown wild-type strain, Sp245, grown at three nutritional conditions (N1, N2 and N3) were harvested by centrifuging 10 ml culture at 5000×g at 4 °C for 10 min. The bacterial pellet was resuspended in 1.5 ml sterile distilled water and the cell suspension was transferred to a pre-weighed 2 ml eppendorf tube. The cells were pelleted by centrifuging at 10,000×g at 4 °C for 2 min. The cell pellet was stored overnight at − 20 °C and subsequently lyophilized (up to 16 h). The dry cell weight was measured to normalize the amount of PHB, to account for the variation caused by the culture growth. The dry cell pellet was transferred into a borosilicate glass tube and crushed using a spatula after addition of 1 ml concentrated H_2_SO_4_. The glass tube was incubated for 30 min at 90 °C in an oven and subsequently cooled on ice. Four ml 7 mM H_2_SO_4_ was added to the tube and mixed by vortexing. The sample was diluted 2-folds, filter-sterilized and 10 μl aliquot was analysed using HPLC system (Shimadzu, Kyoto, Japan). The sample was analysed based on crotonic acid standards (Sigma-Aldrich, USA), in 30% acetonitrile in water (pH 2.8; set with H_3_PO_4_) in a reverse-phase column [Luna® 5 μm C18(2) 100 Å, 250 mm × 4.60 mm, Phenomenex, USA] at a flow rate of 0.5 ml/min at 210 nm with a UV detector.

Biofilm formation was measured by crystal violet binding assay using the microtitre plate [77]. The bacterial strain was grown overnight in LB medium and 1% of it was sub-cultured in 10 ml SSM medium. From this, 100 μl culture was pipetted in a fresh 96-well microtiter plate, the plate was covered and incubated at 30 °C for 48 h, under static conditions. Post incubation, the wells were washed thoroughly with sterile, distilled water to remove planktonic bacteria. To each well, 125 μl of 0.1% crystal violet was added and the stain was removed after 10 min of incubation at RT. The plate was air-dried and 200 μL of 95% ethyl alcohol was added to each stained well to solubilize the dye. The contents of each well were mixed and 125 μL of the crystal violet/ ethyl alcohol solution was transferred to a fresh microtitre plate. The absorbance at 560 nm was measured using Synergy H1 microplate reader (Biotek, USA). All values were normalized with cellular OD_560_ and hence the biofilm formation was represented as OD_560_/cellular OD_560_.

To estimate the nitrogenase activity, acetylene reduction assay [78] and ethylene produced by the bacteria was used as an indirect method since acetylene is a competitive inhibitor of the nitrogenase enzyme activity. Based on this, glass test tubes (30 mL) containing 5 mL of modified SSM media (half- nitrogen and minus nitrogen; C/2 + N/2, C + N/2, C-N) were inoculated with 10 μL of adjusted cell suspension. The headspace in the test tube was 25 ml. The bacterial cells were grown in the 3 nutrient conditions at 30 °C for 48 h (time required to grow the pellicle), the tubes were sealed with a rubber stopper and 1 mL of acetylene gas was injected into the tubes. Gas samples were removed after an incubation time of 1 h and assayed for ethylene using a gas chromatograph, equipped with a flame ionization detector and a Porapak N column. The amount of ethylene produced by the bacterial strains was calculated using the ethylene standards and the final amount of gas in 25 ml headspace subsequently obtained. Bacterial cells grown in SSM media (C + N) were used as control for comparison.

### RNA sequencing

#### Total RNA extraction

The wild-type strain Sp245 was grown under two nutritional conditions: control (VC - SSM with the full strength of nitrogen, N1) and stress-inducing (VN - SSM with half strength of nitrogen, N3). The two samples were labelled as VC and VN, respectively, during the study. Total RNA was extracted using the Ambion PureLink RNA Mini kit (Invitrogen) as per the manufacturer’s instructions with certain modifications (for homogenization of the cell lysate, the lysate was centrifuged at 8000 rpm for 2 min and subsequently proceeded for binding as described in the kit manual). The RNA quantity and quality were determined using the Qubit® Fluorometer and Bioanalyzer (Agilent).

### cDNA library preparationg and next-generation sequencing (RNA-seq)

sRNA libraries were constructed for sequencing according to the Illumina TruSeq Small RNA library protocol outlined in “TruSeq Small RNA Sample Preparation Guide” (Part#15004197Rev.G- December 2014). The prepared library was quantified using Qubit Fluorometer and validated for quality by running an aliquot on the high sensitivity Bioanalyzer Chip (Agilent). The cDNA library was proceeded for sRNA sequencing on an Illumina NextSeq500 platform (Illumina, USA). The sRNA-seq data for both samples, VC and VN, have been submitted to the National Center for Biotechnology Information (NCBI) Gene Expression Omnibus (GEO accession number GSE117764).

### RNA-Seq data analysis

The computational analysis of the RNA-seq output data was carried out as described previously [79] with a few modifications. The quality of the sequencing reads (Fastq output file) was checked using FastQC (Version 0.11.5). The sequences corresponding to the Illumina small 5′ and 3′ adapters (GATCGTCGGACT and TGGAATTCTCGG) were trimmed using CutAdapt [80] and additional filtering was carried out with Trimmomatic (Version 0.36) [81] using default parameters of the software. The quality of the processed output files for both the samples (VC and VN) was again checked using FastQC [82]. Reference-based alignment of the output reads was performed individually for both the samples with Bowtie 2.1.0 [38] using *A. brasilense* Sp245 (GenBank Assembly accession: GCA_000237365.1) as the reference genome. The mapped reads obtained in the BAM files were filtered on the basis of size i.e.; 50-500 bp using an in-house shell script. The data was then analysed for differential gene expression between VC and VN samples by using Cufflinks (v2.1.1 suite pipeline). Cuffmerge and Cuffdiff tools were used to estimate the transcript abundances and differential gene expression. Cufflinks analysis produced scaled expression in the form of fragments per kilobase per million (FPKM). The statistical significance of the differences in FPKM values in the samples was assessed using the paired Wilcoxon test.

### *In-silico* prediction of novel sRNAs

sRNAscanner [83] identifies intergenic sRNA transcriptional units in completely sequenced bacterial genomes based on the transcriptional signals. This tool was used to predict sRNAs restricted to the intergenic regions in *A. brasilense* Sp245 genome with all the parameters set at default values, i.e. 3 provided input matrices: 35box_sRNA.matrix (cut-off: 2), 10box_sRNA.matrix (cut-off: 2), terminator.txt.matrix (cut-off: 3); spacer range between [− 35] & [− 10] promoter boxes: 12–18; unique hit value: 200; minimum cumulative sum of score (CSS): 14 and sRNA length for prediction: 40–350 nucleotides.

### mRNA target prediction

IntaRNAv2.0, [44] facilitates the process of putative target prediction in bacteria, based on features such as conservation of the sRNA and its accessibility, of the mRNA and hybridization energy. The mRNA targets of the validated sRNAs were predicted using IntaRNAv2.0, with default parameters, i.e. nucleotides (NTs) upstream: 75, NTs Downstream: 75, seed length: 7, sRNA folding window size: 150, and *P*-value threshold set at 0.05.

### Prediction of secondary structure and homology search

The secondary structure of the sRNAs was predicted using Mfold web server [84] with default folding temperature of 37 °C. Homology search was carried out for the predicted sRNAs in Bacterial Small Regulatory RNA Database (BSRD) [85] and Rfam database [86]. sRNA with no known homologs were considered as novel sRNAs.

### Conserved motifs and promoter prediction

The conserved motif prediction was carried out using MEME Suite [87] which discovers the novel, ungapped motifs in the nucleotide sequence. Putative promoters and terminator sequences in the predicted sRNA genes were identified using BPROM [43] and FindTerm [88] (software available from SoftBerry), respectively, using default parameters (energy threshold value for FindTerm was − 11). BPROM is a bacterial σ70 promoter recognition program having high accuracy and specificity while FindTerm recognizes the rho-independent terminators.

### sRNA enrichment and northern blot analysis

sRNA enrichment of the isolated RNA preparation from wild-type bacterium *A. brasilense* Sp245 was carried out by adding 5% PEG and 0.5 M NaCl to the sample and incubated overnight at − 80 °C. The sample was centrifuged at maximum speed for 10 min and to the supernatant, 2.5 volume ethanol (100%) and one-tenth volume of 3 M sodium acetate was added. After centrifugation at maximum speed for 30 min, the supernatant was discarded, and the pellet was washed with 1 ml ethanol (80%). Post drying, the pellet was resuspended in 10 μl DEPC water.

The sequences of the probes used for sRNA candidate detection and validation are listed in Additional File 3. The probes were labelled using Biotin DecaLabel DNA Labeling Kit (ThermoFisher Scientific). After electrophoresis on 6% polyacrylamide gel containing 6 M urea, enriched RNA (50 μg in each lane) was electrotransferred onto the nitrocellulose membrane. Specific transcripts on the membranes were detected using Biotin Chromogenic Detection kit (ThermoFisher Scientific) according to the manufacturer’s instructions.

### Quantitative real-time PCRg and expression validation

Gene expression quantification was performed on a CFX-96 Touch, Real-time PCR detection system (Bio-Rad Labs, Inc.) with 50 ng cDNA template using 2x Sso Fast Eva Green Supermix Dye (Bio-Rad Labs, Inc.) with the manufacturer recommended reaction set-up. The thermal cycling conditions were optimized as per the primers designed for each sRNA and shown in Additional File 7**.** In each analysis, a No Template Control (NTC) was included and each sample was set up in triplicate. Each plate was repeated at least thrice. Relative gene expression study by qPCR was performed using 16S rDNA as the reference gene for normalization of expression (ΔΔCq) of each sRNA. The data obtained was further reported in expression fold change in control (VC) versus nitrogen starvation (VN) conditions (2^-ΔΔCq, Fig. 4). The data was statistically analysed for variance by ANOVA followed by Duncan’s Post hoc analysis at *P* ≤ 0.05. All analysis was performed with Statistical Package for Social Sciences (SPSS ver. 22.0 for Windows).

## Supplementary information

Additional File 1. Table showing the quality check of the raw sRNA sequencing data files.

Additional File 2. Worksheets showing details of the curated, 3860 candidate sRNAs differentially expressed between samples VC and VN, |the 468 candidate sRNAs that conform to the size requirement of 50-500 bp and the 59 candidate sRNAs from the 468 sRNAs that are significantly, differentially expressed between samples VC and VN.

Additional File 3. Northern Blotting for candidate sRNAs. Table 3A - Probes used for Northern Blotting and Table 3B - Validation of sRNA expression using northern blot analysis.

Additional File 4. Motifs, promoters and terminators predicted for the 59 significantly differentially expressed sRNAs.

Additional File 5. Details of the targets predicted for the 59 significantly expressed sRNAs. The table highlights target details and score for each category of targets for all sRNAs as well as upregulated/downregulated target mRNA details.

Additional File 6. Details of the 14 sRNAs with no InterProScan hits and their respective mRNA target categories and scores.

Additional File 7. Details of Sixteen sRNAs validated by qRT-PCR based differential expression analysis.

## Data Availability

The small RNA-seq data has been submitted to the National Center for Biotechnology Information (NCBI) Gene expression Omnibus (GEO) with accession number GSE117764.

## Abbreviations

sRNA: small RNA
FPKM: Fragments per kilobase transcript per million mapped reads
BSRD: Bacterial Small Regulatory RNA
SAM: Sequence alignment map
RIN: RNA Integrity Number
PHB: Polyhydroxybutyrate
PGPB: Plant Growth-Promoting Bacteria
PAB: Plant associated bacteria
