# Supplementary material for: Genome-wide identification of Azospirillum brasilense Sp245 small RNAs responsive to nitrogen starvation and likely involvement in plant-microbe interactions
Source: BMC Genomics. 2020 Nov 23;21:821. doi: 10.1186/s12864-020-07212-7 (PMC7685610; doi:10.1186/s12864-020-07212-7)
Supplement: Supplementary file 1 — Table showing the quality check of the raw sRNA sequencing data files. [file 12864_2020_7212_MOESM1_ESM.docx]

**SUPPLEMENTARY FILES**

**Additional File 1**: Quality check of the raw sRNA sequencing data files.

| **Sample_name** | **VC_R1**^a^ | **VC_R2**^b^ | **VN_R1**^a^ | **VN_R2**^b^ |
| --- | --- | --- | --- | --- |
| Total_number_of_bases | 2500226743 | 2500226743 | 4107297093 | 4107297093 |
| Total_number_of_reads | 16557793 | 16557793 | 27200643 | 27200643 |
| %_bases_>=Q20 | 93.68 | 89.07 | 94.02 | 89.91 |
| %_bases_>=Q30 | 90.98 | 84.88 | 91.36 | 86.18 |
| Average_read_length | 151 | 151 | 151 | 151 |
| Max_read_length | 151 | 151 | 151 | 151 |
| Min_read_length | 151 | 151 | 151 | 151 |
| Number_of_base_A | 435262144 | 408208022 | 734502131 | 671241263 |
| Number_of_base_T | 390180937 | 360672790 | 610358575 | 576771451 |
| Number_of_base_G | 1195768531 | 1318940570 | 2046469896 | 2251429162 |
| Number_of_base_C | 478691537 | 409420385 | 715163546 | 600913088 |
| Number_of_base_N | 323594 | 2984976 | 802945 | 6942129 |
| GC_content_% | 66.97 | 69.13 | 67.24 | 69.45 |

^a^VC_R1 and VN_R1 represent the forward reads for non-stressed (VC) and stressed (VN) samples, respectively.

^b^VC_R2 and VN_R2 represent the reverse reads for non-stressed (VC) and stressed (VN) samples respectively.

Note: The RNA concentration and RNA Integrity Number (RIN) for sample VC was 979ng/µL and 10, respectively; and for sample VN was 1447.6ng/ µL and 9.5, respectively.

RIN values depict the quality of the RNA and range from 10 (intact) to 1 (totally degraded).
