# Supplementary material for: Genome-wide identification of Azospirillum brasilense Sp245 small RNAs responsive to nitrogen starvation and likely involvement in plant-microbe interactions
Source: BMC Genomics. 2020 Nov 23;21:821. doi: 10.1186/s12864-020-07212-7 (PMC7685610; doi:10.1186/s12864-020-07212-7)

**Additional File 3A**: Probes used for northern blotting validation of *Azospirillum brasilense* sRNAs in this study.

| **sRNA candidate** | **Probe Sequence** |
| --- | --- |
| AbSp_4 | ACCTCGCCCACTTCCCTTAGCTTGGGAGCAGCATTCGAACCCTTCG |
| AbSp_22 | GGACCACCAGTACCGCTCCGAGGGTTGTGGGCTAGGGTAAGGCTTGA |
| AbSp_13 | CGTCGGCATTGCTCAAAGCCGAGCCCAGCAAGCAGGTCGGAGGGTG |
| AbSp_153 | CAGTCAGTGGCGGCAATCCGCCGTTGCCGCTGGCCTCGTTGCAACAGCAACCGTTGATAACGTTACCGG |
| AbSp_459 | AGTGCGAATAGTTCGCGCGCGAGATTGGTTGACTCGATGATCG |
| AbSp_460 | AAGCAGTCCAGCGTTATTAGGGGTCCCGGCTATTAGAATCTGAGAGCCCTCGACAG |
| AbSp_461 | CGCGGGCATCGAGTCGACCTATCTCGTGGTCTGATGCTTAGAC |
| AbSp_462 | CTACCCACCGGCTCGCCAAATTCCGAGGTCAGAACTTTTGACCGCACCCCCGTTGAGGTGGCACCCAAGCTTAG |
| AbSp_463 | GCCTAATCGAGTCAGCCATCTCGTCGCCTGACAATTAGGCGCACAGCGACCAAGCTCAGGTCGTAG |
| AbSp_464 | GATCATCGAGTCAACCAATCTCGCGCGCGAACTATTCGCACTCCAGCCTC |
| AbSp_466 | ACTGTTACGTTTCTGGCAGAGGAAAAGGCGTTAGTTTGTTCTTCTTTTACGGGAGCG |
| AbSp_465 | ACACAAAATTTACCTACACGCTTAAGATAACAAACAATAGTCGCCAAAC |

**Additional File 3B:** Validation of sRNA expression using northern blot analysis of the selected sRNA candidates retrieved from RNA-seq and *in-silico* study. Arrows indicate predominant bands. The size of the sRNA was estimated by comparing to GeneRuler Low Range ladder (ThermoFisher Scientific, USA), which is indicated on left of each picture.


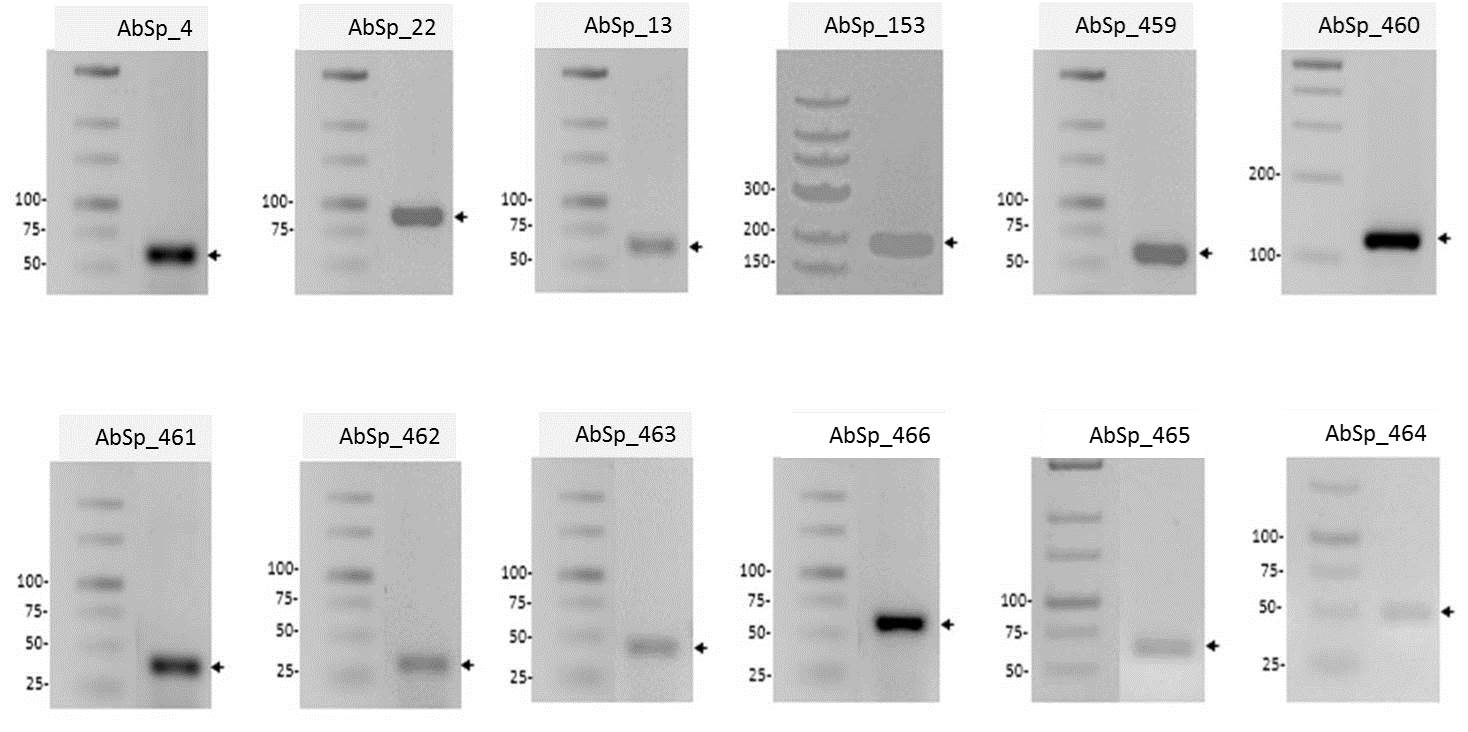

Supplement: Supplementary file 3 — Northern Blotting for candidate sRNAs. Table 3A - Probes used for Northern Blotting and Table 3B - Validation of sRNA expression using northern blot analysis. [file 12864_2020_7212_MOESM3_ESM.docx]
